# Complete absence of M2-pyruvate kinase expression in benign pancreatic ductal epithelium and pancreaticobiliary and duodenal neoplasia

**DOI:** 10.1186/1471-2407-9-327

**Published:** 2009-09-15

**Authors:** Mark M Aloysius, Abed M Zaitoun, Timothy E Bates, Abdulkader Albasri, Mohammad Ilyas, Brian J Rowlands, Dileep N Lobo

**Affiliations:** 1 Division of Gastrointestinal Surgery, Nottingham Digestive Diseases Centre NIHR Biomedical Research Unit, Nottingham University Hospitals, Queen's Medical Centre, Nottingham NG7 2UH, UK; 2 Department of Pathology, Nottingham Digestive Diseases Centre NIHR Biomedical Research Unit, Nottingham University Hospitals, Queen's Medical Centre, Nottingham NG7 2UH, UK; 3 Department of Community Health Sciences, University of Nottingham, Nottingham NG7 2UH, UK

## Abstract

**Background:**

Elevated serum concentrations of M2-pyruvate kinase (M2-PK) correlate with poor prognosis in patients with pancreaticobiliary and duodenal cancer, but the expression of M2-PK in formalin-fixed pancreatic tissue is unknown. We aimed to characterise the immunohistochemical expression of M2-PK in archived specimens of pancreaticobiliary and duodenal cancers, premalignant lesions, chronic pancreatitis, and normal pancreas.

**Methods:**

Immunohistochemical staining was performed with mouse anti-M2-PK monoclonal antibody (clone DF-4) at an optimal dilution of 1:25 on tissue microarrays constructed from formalin-fixed paraffin-embedded pancreatic tissue of 126 consecutive patients undergoing pancreatic resections between June 2001 and June 2006. 104 underwent resection for cancer and 22 for chronic pancreatitis. 78 specimens of chronic pancreatitis tissue were obtained adjacent to areas of cancer. Normal pancreatic tissue was obtained from the resection specimens in a total of 30 patients. Metastatic tumours in 61 regional lymph nodes from 61 patients were also studied. A further 11 premalignant pancreaticobiliary and duodenal lesions were studied. M2-PK expression was quantified with the immunohistochemical score (IHS; Range 0-12).

**Results:**

Benign non-ductal tissue in chronic pancreatitis and normal pancreas showed variable expression of M2-PK (IHS = 1 in 25%, IHS = 2-3 in 40%, IHS>3 in 40%). Benign pancreatic ductal epithelium, all primary pancreaticobiliary and duodenal premalignant lesions and cancers (and lymph node metastasis) showed complete lack of expression (IHS = 0).

**Conclusion:**

Complete lack of M2-PK expression was observed in benign pancreatic ducts, premalignant lesions and cancer. M2-PK is present only in benign non-ductal epithelium in normal pancreas and peri-tumoural tissue.

## Background

The enzyme pyruvate kinase (PK) plays a central role in aerobic glycolysis, a metabolic process that is increased in tumour cells [1]. During carcinogenesis, the expression of tissue specific pyruvate kinases, such as M1-PK (muscle and brain), L-PK (liver and kidney), and R-PK (red blood cells) is altered and, in addition, the M2-PK isoform, normally found in embryonic tissue, is also expressed [2,3]. Pyruvate kinase isoenzymes normally exist as highly active tetramers. However, the tetrameric M2-PK can change to an inactive dimeric form, which is predominant in tumour cells, and has a low affinity for phosphoenolpyruvate and also interacts with oncoproteins [4]. The predominance of the inactive dimeric form in tumour cells leads to an accumulation of all phosphometabolites preceding pyruvate kinase in the glycolytic pathway, such as phosphoenolpyruvate, glycerate 3-phosphate, glyceraldehyde 3- phosphate, fructose 1,6-bisphosphate, ribose-5 phosphate and 5'-ribose-pyrophosphate. These are then available as precursors for synthesis of nucleic acids, amino acids and phospholipids [5,6]. This switch in metabolism may allow tumour cells to invade areas with low concentrations of oxygen and glucose. Tumour M2-PK has been detected in normal fresh colonic epithelium, but increases greatly in colonic adenocarcinomas, and plasma concentrations correlate with tumour load in patients with lung cancer [1,3]. In addition, raised concentrations of tumour M2-PK correlate with the ability of renal carcinoma cells to metastasise [7]. Recently, tumour M2-PK has been shown to be measurable not only in plasma, but also in faeces, leading to an interest in its potential as a metabolic marker for the screening of patients at increased risk of colorectal and gastric cancer [8,9].

Elevated serum concentrations of M2-PK have been found to correlate with poor prognosis in patients with pancreaticobiliary and duodenal cancer [10]. A pilot study by Mottet *et al. *[11] has shown moderate presence of M2-PK in acetone fixed tissue. There are, however, no published studies on tissue expression of M2-PK in these tumours in formalin-fixed tissue. We sought to evaluate the immunohistochemical expression of M2-PK in premalignant tissue and cancers of pancreaticobiliary and duodenal origin and compare this with expression in chronic pancreatitis and in normal pancreatic tissue.

## Methods

### Study design and setting

This was an immunohistochemical study on archived formalin-fixed pancreatic tissue from all patients who underwent pancreatic resections between June 2001 and June 2006 at a university teaching hospital.

### Patients

We studied 126 consecutive patients who underwent pancreatic resections for malignant and benign disease. A further 11 patients who underwent biopsies or resections for premalignant lesions of pancreaticobiliary and duodenal origin were also studied. Specimens of chronic pancreatitis tissue were obtained from those who underwent resection for chronic pancreatitis and from areas of chronic pancreatitis that were adjacent to cancers. Normal pancreatic tissue was obtained from the resection specimens, adjacent to areas of cancer or chronic pancreatitis. Metastatic lymph nodes from patients with cancer were also studied.

### Construction of tissue microarrays

Formalin-fixed paraffin-embedded tissue blocks containing pancreatic ductal adenocarcinoma, ampullary adenocarcinoma, cholangiocarcinoma, duodenal adenocarcinoma, chronic pancreatitis and normal pancreatic tissue were identified on hematoxylin and eosin stained slides and marked by a single gastrointestinal histopathologist (AMZ). The marked areas in the corresponding paraffin blocks (donor blocks) were used for the construction of tissue microarrays. From these defined areas of each specimen, triplicate tissue cores with a diameter of 0.6 mm were taken from donor block and arrayed into a recipient paraffin block using a tissue puncher/arrayer (Beecher Instruments, Silver Spring, MD, USA) as previously described [12]. It has been shown that tissue microarray cores in triplicate provide a sufficient level of sampling to give an excellent representation of any block [13,14]. 5 μm sections of the tissue array block were cut and placed on Fisherbrand Colorfrost/Plus microscope slides (Fisher Scientific, Pittsburgh, PA) for immunohistochemical staining.

### Immunohistochemical staining

**Figure 1:**
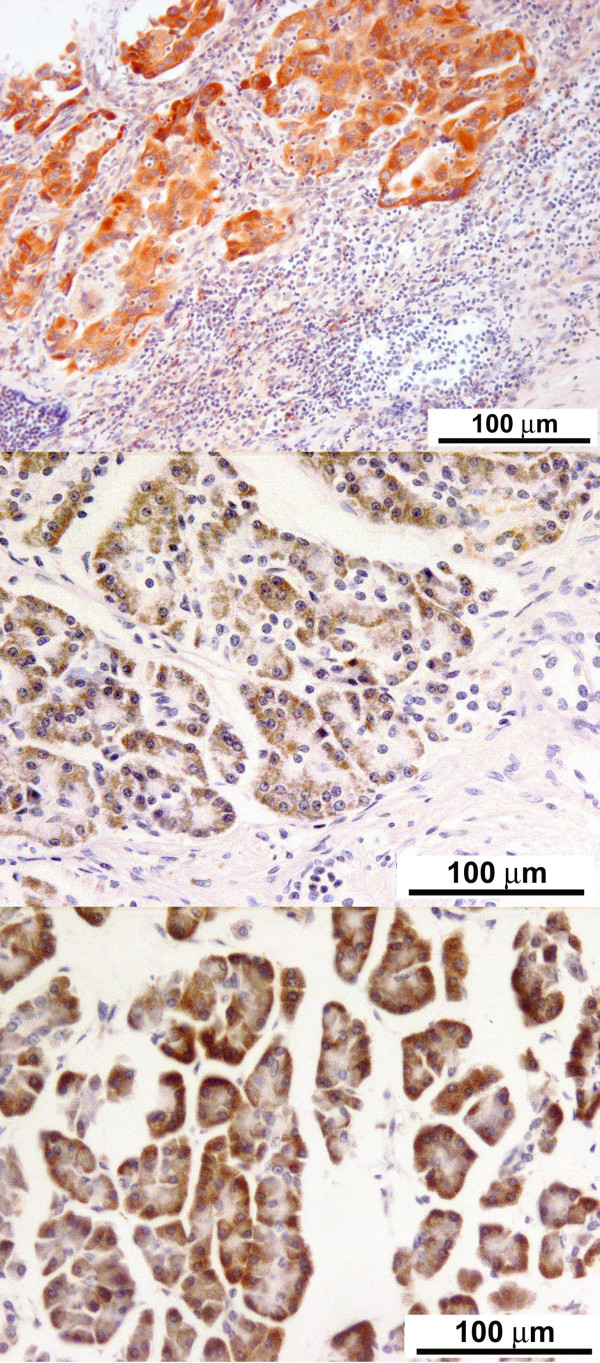
**Strong expression of M2-pyruvate kinase in a positive control (lung cancer) and benign pancreatic non-ductal epithelium**: Immunohistochemical staining for M2-pyruvate kinase on formalin fixed lung adenocarcinoma (× 20), demonstrating a strong uptake of the stain. This was used as a positive control (top panel); Immunohistochemical staining for M2-pyruvate kinase on chronic pancreatitis (× 20), demonstrating moderate staining of benign non-ductal pancreatic epithelium (middle panel); Immunohistochemical staining for M2-pyruvate kinase on normal non-ductal pancreatic epithelium (× 20), demonstrating a strong uptake of stain (bottom panel).

Slides were deparaffinised in xylene, then hydrated via graded dilutions of alcohol followed by running tap water. Following a rinse in deionized water, the sections were incubated with Proteinase-K (S3020; Dako UK Ltd., Ely, UK) for 15 min at room temperature to unmask antigenicity, and subsequently a Labelled Streptavidin Biotin (LSAB) immunoperoxidase procedure was performed using 3,3'-diaminobenzidine (DAB) as the chromogen on a TechMate 500+ automated stainer (Dako). The staining protocol consisted of incubating the sections with 0.3% hydrogen peroxide for 10 min to block endogenous peroxidases, washing in phosphate buffered saline (PBS) buffer, followed by incubation in normal goat serum for 20 minutes. The slides were washed then incubated in mouse anti-M2-PK monoclonal antibody, (clone DF-4) from ScheBo^® ^Biotech (Giessen, Germany) at an optimal dilution of 1:25 (optimisation was achieved using a range of dilutions from 1:10 to 1:50) in a standard antibody diluent (S2022; Dako, UK) for 1 hr at room temperature, followed by further washing in Phosphate Buffer Saline (PBS). Sections were then incubated with a biotin linked secondary antibody (30 min) followed by washing in PBS and streptavidin-horseradish peroxidase (30 min) using reagents in a kit from Dako (K5001). Immunostaining was visualized using DAB for 10 minutes, also present in this kit followed by light counterstaining with haematoxylin. Two types of negative control experiments were performed on the same TMAs omitting the incubation with the primary (anti-M2-PK) antibody and the Proteinase K antigen extraction steps. The TMAs did not stain positively in either negative control experiment, confirming the positive staining observed using this protocol was that of M2-PK. Adenocarcinoma of the lung was used as a positive control (Figure 1, top panel) and pancreatic islets as negative controls. Whole tissue sections of pancreatic tumours (n = 5) were also stained by this protocol.

### Immunohistochemical scoring

Immunostaining was reviewed by AMZ and AMM independently and the average score taken. There was no inter-observer variability (i.e. 100% concurrence) for negative staining. However, for positive staining the inter-observer variability was 2.9%. The level of M2-PK expression was calculated by combining an estimate of the percentage of immunoreactive cells (quantity score) with an estimate of the staining intensity (staining intensity score) as follows. No staining was scored as 0, 1-10% of cells with positive staining were scored as 1, 10-50% as 2, 50-70% as 3, and 70-100% as 4. Staining intensity was rated on a scale of 0 to 3 as follows: 0 = negative (no colour); 1 = weak brown, 2 = moderate brown, and 3 = strong brown. The raw data were converted to the immunohistochemical score (IHS) by multiplying the quantity and staining intensity scores. Therefore, the score could range from 0 to 12 [15].

### Ethics

The conduct of this study was approved by the Ethics Committee of Nottingham University Hospitals.

## Results

The median (IQR) age of the 77 male and 49 female patients was 63 (56-71) years. Of the 126 consecutive patients who underwent pancreatic resections 104 had histologically confirmed pancreaticobiliary and duodenal cancer and 22 had chronic pancreatitis. Specimens of chronic pancreatitis tissue were obtained from areas adjacent the cancer in a further 78 patients. Normal pancreatic tissue was obtained from the resection specimens, adjacent to areas of cancer or chronic pancreatitis in a total of 30 patients. Sixty one metastatic lymph nodes from 61 patients were also studied. The premalignant lesions studied were pancreatic intraepithelial neoplasia-I (Pan IN-I) *(n = 1)*, Pan IN-III *(n = 1)*, ampullary adenoma with low grade dysplasia *(n = 2)*, ampullary adenoma with high grade dysplasia *(n = 2)*, ampullary intraductal low grade dysplasia *(n = 1)*, pancreatic intraductal low grade dysplasia (n = 1), common bile duct with low grade dysplasia *(n = 1)*, common bile duct with low grade tubular adenoma *(n = 1)*, duodenal tubular adenoma with high grade dysplasia *(n = 1)*.

**Figure 2:**
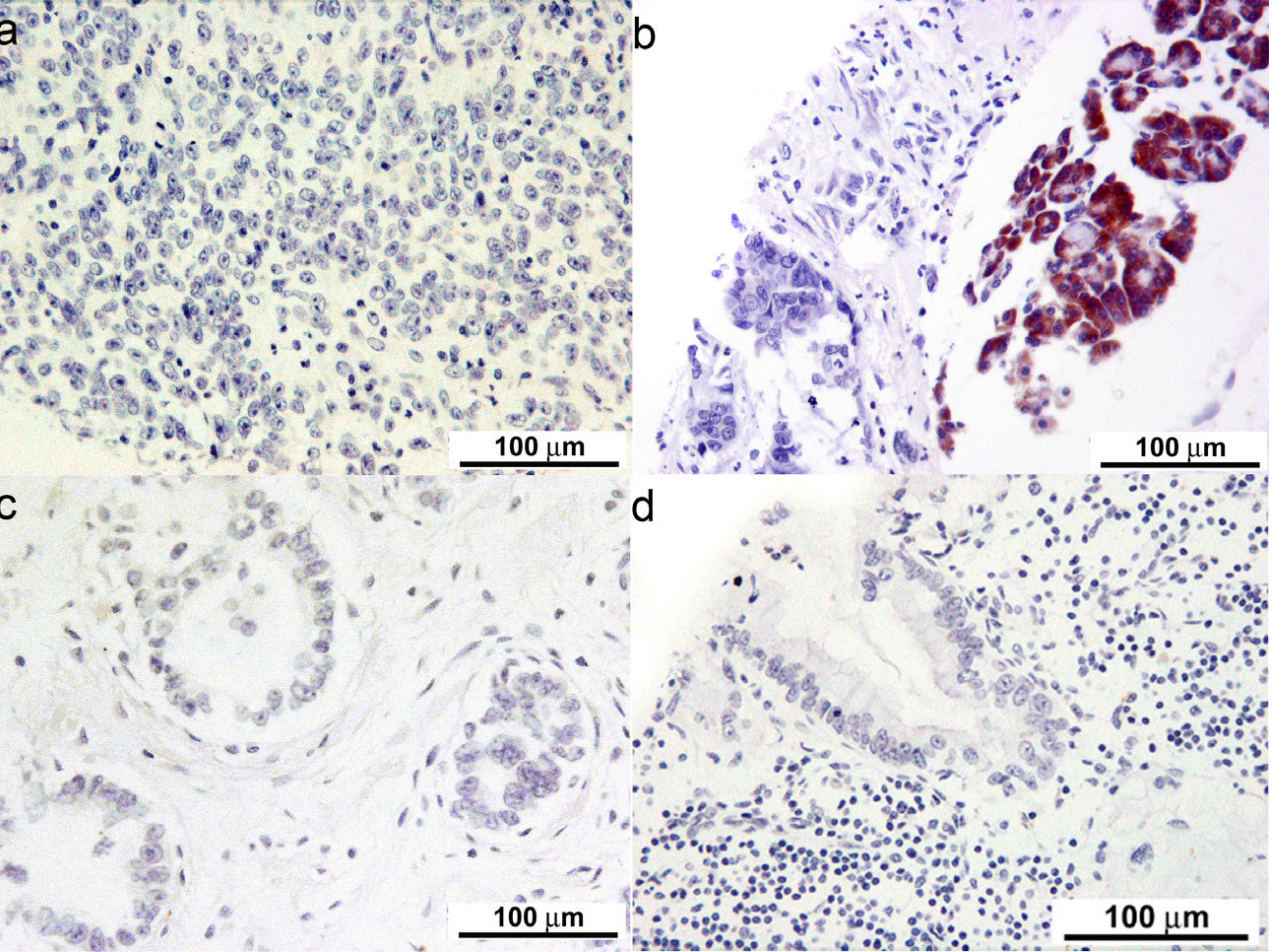
**Lack of expression of M2-pyruvate kinase in duct-derived cancers**: (a) Immunohistochemical staining for M2-pyruvate kinase on diffuse anaplastic pancreatic ductal adenocarcinoma (× 20), demonstrating a complete lack of staining in tumour epithelium; (b) Immunohistochemical staining for M2-pyruvate kinase on poorly differentiated pancreatic ductal adenocarcinoma, with adjacent chronic pancreatitis (× 20), demonstrating a complete lack of staining in the tumour, but intense staining in the benign non-ductal reactive epithelium; (c) Immunohistochemical staining for M2-pyruvate kinase on well differentiated adenocarcinoma pancreas (× 20); (d) Immunohistochemical staining for M2-pyruvate kinase on metastatic pancreatic ductal adenocarcinoma in the regional lymph node (× 20), demonstrating a complete lack of staining in the tumour epithelium and lymph node.

**Figure 3:**
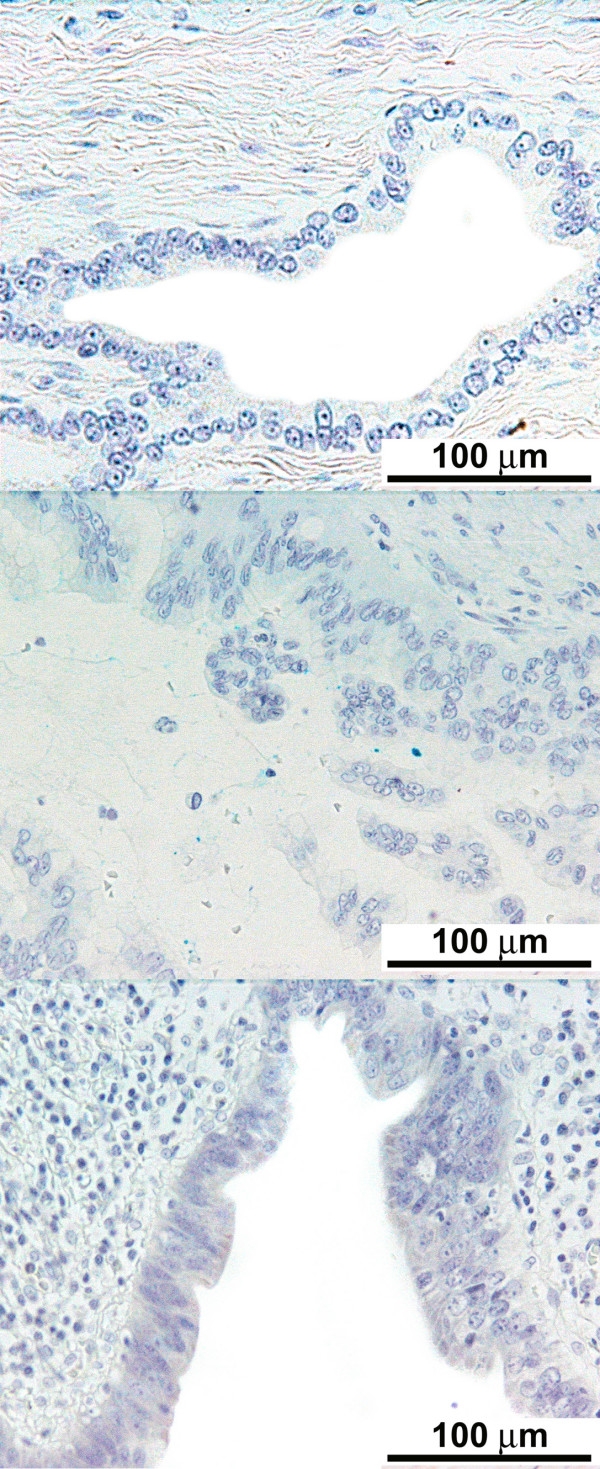
**Lack of expression of M2-pyruvate kinase in premalignant pancreaticobiliary epithelium**: Premalignant lesions showing lack of M2-PK expression pancreatic intraepithelial neoplasia I (× 20) (top panel); pancreatic intraepithelial neoplasia III (× 20) (middle panel); common bile duct with low-grade dysplasia (× 20) (bottom panel).

The degree of differentiation of various cancers and lymph node involvement are described in Table 1. The immunohistochemical scores of the stained tissue are shown in Tables 2 and 3. There was consistent lack of expression of M2-PK (IHS = 0) in all pancreaticobiliary and duodenal tumours, irrespective of tumour type or differentiation (Figure 2a-c). Similarly, metastatic pancreaticobiliary and duodenal tumours in regional lymph nodes lacked the expression of M2-PK (Figure 2d). However, chronic pancreatitis tissue and normal pancreatic tissue around these tumours were found to have a variable cytoplasmic expression (IHS = 1-12) of M2-PK in non-ductal tissue, but benign ductal epithelium lacked complete expression (Figure 1 middle and bottom panel). All premalignant lesions of pancreaticobiliary and duodenal origin also showed complete lack of expression of M2-PK (Figure 3). The staining pattern seen in the tissue microarrays was similar to that seen in whole tissue sections.

**Table 1:** Tumour type and differentiation for the 104 pancreatic resections for cancer

	Pancreatic ductal adenocarcinoma (n = 44)	Ampullary adenocarcinoma (n = 31)	Cholangiocarcinoma (n = 24)	Duodenal adenocarcinoma (n = 5)
Well differentiated	5	1	7	0
Moderately differentiated	29	18	17	3
Poorly differentiated	10	5	7	2
Patients with metastatic lymph nodes	26	12	20	3

**Table 2:** Immunohistochemical scoring (IHS) of the expression of M2-PK in malignant and benign pancreatic tissue microarrays.

TISSUE (n)	IMMUNOHISTOCHEMICAL SCORE [n (%)]						
	0	1	2-3	4-5	6-7	8-9	10-12
**Pancreatic ductal adenocarcinoma (44)**	44 (100)						
Tumour in lymph nodes (26)	26 (100)						
Chronic pancreatitis (36)		2 (6)	30 (83)	4 (11)			
Normal pancreas (8)			7 (87.5)			1(12.5)	
							
**Ampullary adenocarcinoma (31)**	31 (100)						
Tumour in lymph nodes (20)	20 (100)						
Chronic pancreatitis (26)	9 (34.6)		6 (23)	7 (27)		4 (15.4)	9 (34.6)
Normal pancreas (8)				2 (25)	4(50)	1 (12.5)	1 (12.5)
							
**Cholangiocarcinoma (24)**	24 (100)						
Tumour in lymph nodes (12)	12(100)						
Chronic pancreatitis (11)		2 (18.1)	5 (45.5)	4 (36.4)			
Normal pancreas (11)			5 (45.5)	5 (45.5)	1 (9)		
							
**Duodenal adenocarcinoma (5)**	5 (100)						
Tumour in lymph nodes (3)	3 (100)						
Chronic pancreatitis (5)			4 (80)		1 (20)		
Normal pancreas (1)				1 (100)			
							
**Chronic pancreatitis (22)**		12 (54.5)		8 (36.5)	2 (9)		
Normal pancreas around pancreatitis (2)				1 (50)	1 (50)		

**Table 3:** Summary of M2-PK expression in benign and malignant pancreatic tissue.

TISSUE (n)	IMMUNOHISTOCHEMICAL SCORE [n (%)]						
	0	1	2-3	4-5	6-7	8-9	10-12
**Primary adenocarcinoma (104)**	104 (100)						
**Regional lymph nodes (tumour +ve 61)**	61 (100)						
**Regional lymph nodes (tumour -ve 39)**	39 (100)						
**Chronic pancreatitis (100)**		25 (25)	39 (39)	22 (22)	10 (10)		4 (4)
**Normal pancreas (30)**			12 (40)	9 (30)	6 (20)	2 (7)	1 (3)

## Discussion

M2-PK is thought to be a serum marker for a variety of cancers [10,16-23] and its serum concentrations in patients with pancreatic cancer correlate with disease burden and prognosis [10]. Furthermore, a previous study showed serum M2-PK levels to be elevated not only in pancreatic cancer but also in benign pancreatic disease [24], suggesting a more complex mechanism of expression. Since the tissue expression of M2-PK in pancreaticobiliary and duodenal cancers was previously unknown, we sought to examine this using immunohistochemistry. Quite unexpectedly, we found complete lack of expression of M2-PK in every case of pancreaticobiliary and duodenal cancer examined irrespective of type and differentiation. In contrast, varying degrees of expression of M2-PK were observed in all normal non-ductal pancreatic tissue and chronic pancreatitis, but benign pancreatic ducts lacked expression. All premalignant lesions of pancreaticobiliary and duodenal origin also showed complete lack of expression of M2-PK. It appears that although pancreatic acinar tissue expresses M2-PK, the lack of M2-PK expression is a common immunohistochemical feature of normal pancreatic ductal epithelium and of dysplastic or neoplastic change in pancreaticobiliary and duodenal tissue, which, to the best of our knowledge, has not been reported previously.

The lack of staining observed in our study could have been due to undersampling, as we only examined TMA cores. However, this is unlikely as previous studies [13,14] have shown that three cores are adequate. Moreover, whole tissue sections of pancreatic tumours stained identically to the tissue microarrays also demonstrated lack of M2-PK in pancreatic cancer and benign ductal epithelium. Another reason could be that the antibody epitope that we used may only be staining the active (tetrameric) form of pyruvate kinase, in which case the positive control (lung adenocarcinoma) used in this study should not have stained. Concurrent plasma concentrations of M2-PK could not be evaluated in our series as the study had a retrospective design.

The differential staining observed between benign pancreatic ducts, premalignant ductal lesions, duct-derived cancers and non-ductal epithelium may possibly reflect an altered Warburg effect in the pancreatic ducts (alteration of aerobic glycolysis by which ductal cells survive in hypoxic conditions and produce lactate) and it is quite possible that alternative pathways of pancreatic tumour cell survival may exist which are yet to be unravelled [25]. Such an alteration of aerobic glycolysis in ducts and duct derived cancers may possibly explain their resilience to hypoxia and therefore a survival advantage of cancer cells in metabolically hostile microenvironments.

The antibody that we used was designed only for formalin/acetone fixed pancreatic specimens and not for fresh pancreatic tissue. Our study contrasts with the pilot study by Mottet *et al*. [11], in that their method of antigen retrieval is not clearly explained and they used the same antibody in acetone fixed pancreatic tissue. However, as yet, these data [11] have only been published in abstract form and further details are awaited. Artifactual immunostaining in acetone fixed tissue is well known phenomenon as the fixation occurs through a process of denaturation, by the disruption of hydrophobic bonds which give protein their tertiary structure [26]. The precipitation and aggregation of proteins observed through acetone fixation is a very different process observed from the cross-linking, which occurs with the aldehyde fixatives. Formalin fixation is far more reliable method than acetone fixation in preserving the tertiary antigenic structure of proteins of interest. It is possible that the M2-PK may have degraded, through the process of acetone fixation, in their study and the immunostaining observed could have been a false positive artefact.

Elevated M2-PK concentrations in plasma or serum, are not specific enough to be a screening tool in pancreatic cancer as high levels of this enzyme can also be found in the plasma/serum of patients with pancreatitis and other inflammatory conditions, unrelated to pancreatic pathology [27]. M2-PK may be elevated falsely in patients with jaundice, which may reflect altered biokinetics of its elimination from blood [28]. Moreover, the source of M2-PK detected in the serum has not yet been confirmed to date. Our study may provide a clue that the source may be normal or inflamed pancreatic non-ductal epithelium. Hence, tissue expression of M2-PK may reflect more accurately the underlying pancreatic pathology. It would be of interest to observe if the rare acinar cell pancreatic carcinoma (non-ductal epithelium derived cancer) expresses M2-PK and if true, this is likely to be a positive expression marker for acinar-cell derived tumours.

## Conclusion

In this study, positive immunohistochemical staining for M2-PK was observed only in benign non-ductal epithelium in normal pancreas and peri-tumoural tissue and there was a complete lack of M2-PK expression in benign pancreatic ducts, premalignant lesions and cancer. This finding may help complement the immunohistochemical diagnosis of pancreaticobiliary and duodenal neoplasms.

## Competing interests

The M2-PK antibody was supplied free of charge by ScheBo Biotech, Giessen, Germany.

MMA has received funding from Schebo^® ^Biotech to attend a conference. None of the other authors has a conflict of interest to declare.

## Authors' contributions

MMA was involved with the design of the study, acquisition of data, data analysis and drafting of the manuscript. AMZ was involved with the design of the study, acquisition of data, data interpretation and critical review of the manuscript. TEB, MI and BJR were involved with the design of the study, data interpretation and critical review of the manuscript. AA was involved with acquisition of data and data analysis. DNL was involved with the design of the study, data interpretation, critical review of the manuscript and overall supervision of the work. All authors have read and approved the final version of the manuscript.

## Pre-publication history

The pre-publication history for this paper can be accessed here:

http://www.biomedcentral.com/1471-2407/9/327/prepub
